# Macular findings in Spectral Domain Optical Coherence Tomography and OCT Angiography in a patient with Kearns–Sayre syndrome

**DOI:** 10.1186/s40942-017-0077-8

**Published:** 2017-07-10

**Authors:** Alvaro Ortiz, Juan Arias, Pedro Cárdenas, John Villamil, Marcela Peralta, Luis C. Escaf, Jacobo Ortiz

**Affiliations:** 1 Fundación Oftalmológica de Santander Carlos Ardila Lulle (FOSCAL), Floridablanca, Colombia; 2 Centro Oftalmológico ALJAORZA, Second Floor, Machala, Ecuador

**Keywords:** Kearns–Sayre syndrome, Mitochondrial disease, Optical Coherence Tomography, Retinal pigmentary epithelium

## Abstract

**Background:**

To report the clinical, electrophysiological and the anatomical findings in a patient with Kearns–Sayre syndrome (KSS).

**Case Presentation:**

We present the case of a 55-year-old female with KSS, who developed systemic features and ocular manifestations as ophthalmoplegia and retinal dysfunction, that were corroborated by electrophysiological test and High Definition Spectral Domain Optical Coherence Tomography (HD SD OCT) and OCT-Angiography (OCT-A).

**Conclusion:**

We report a patient with KSS, accompanied by some alterations of the RPE and photoreceptors observed in the external HD SD OCT and OCT-A. In the best of our knowledge, this is the first report in the literature of HD SD OCT findings in a patient with KSS.

## Background

Kearns–Sayre syndrome (KSS) was first described in 1958 [1]. It is a neuromuscular disorder that belongs to a group of genetic diseases, characterized by deletions in mitochondrial DNA (mtDNA) that also includes Pearson syndrome and chronic progressive external ophthalmoplegia (CPEO) [2, 3]. The classic triad include pigmentary retinopathy, CPEO and heart block [1–4], but additional features like cerebellar ataxia, increased cerebrospinal fluid protein level have been described, among others [5, 6].

Symptoms usually appear in adolescence, before the second decade of life, but there are variants of later presentation in adulthood [5, 6]. The diagnosis is made with polymerase chain reaction (PCR) and muscle biopsy [6]. To our knowledge, no High Definition Spectral Domain Optical Coherence Tomography (HD SD OCT) or OCT-Angiography (OCT-A) features in KSS have been described to date. This report shows bilateral tomographical findings in a patient with KSS.

## Case Presentation

**Fig. 1:**
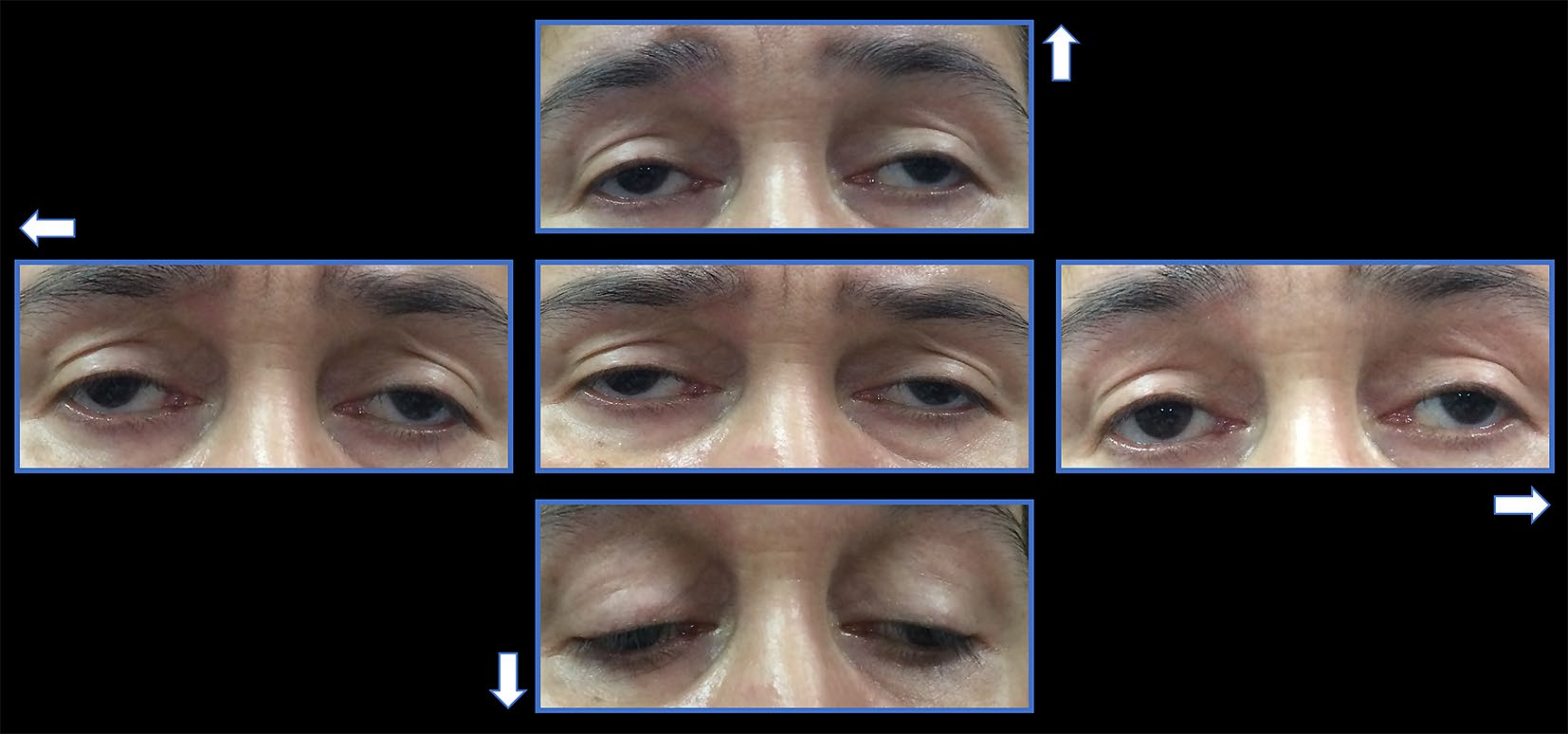
Bilateral palpebral ptosis and motor limitation in all positions of gaze (*Arrows* indicate the position of gaze)

**Fig. 2:**
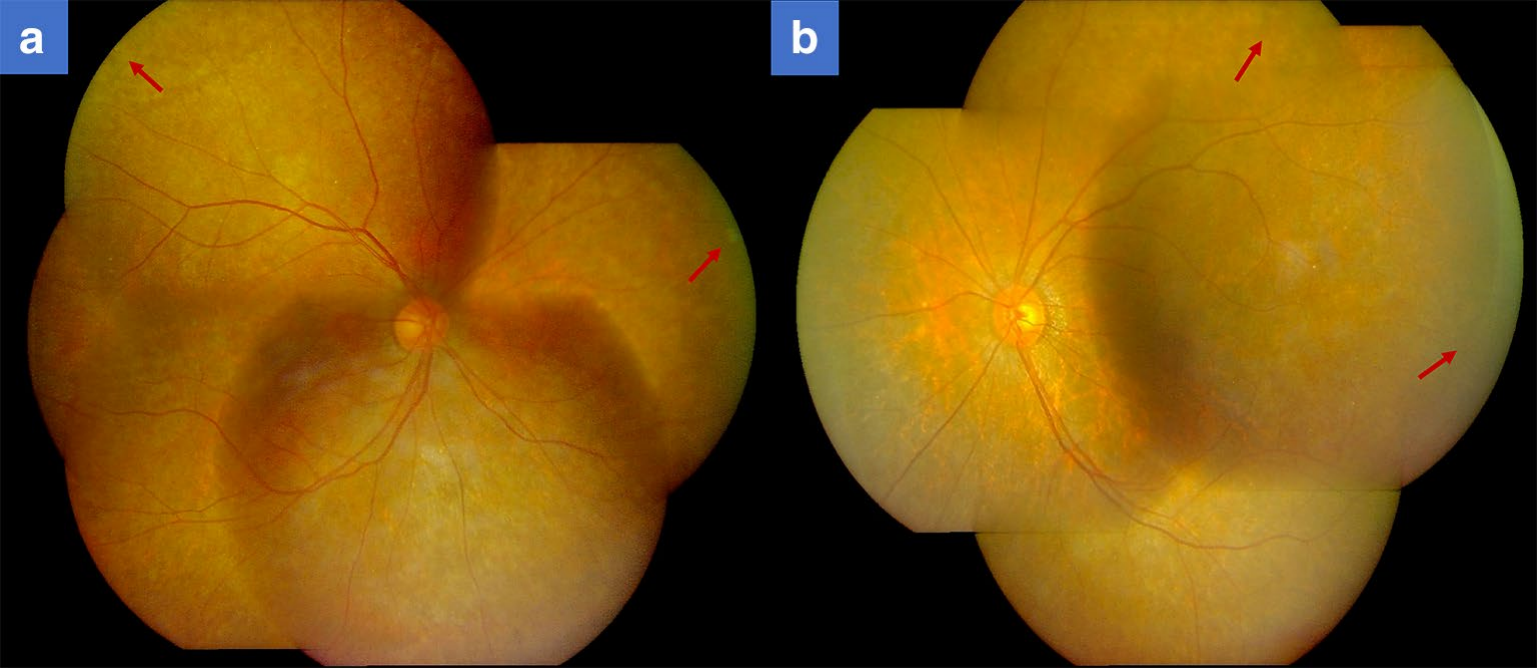
Panoramic color pictures of Fundus showing salt and pepper pattern (The *red arrows* showed the bone spicules). **a** Right eye, **b** left eye

**Fig. 3:**
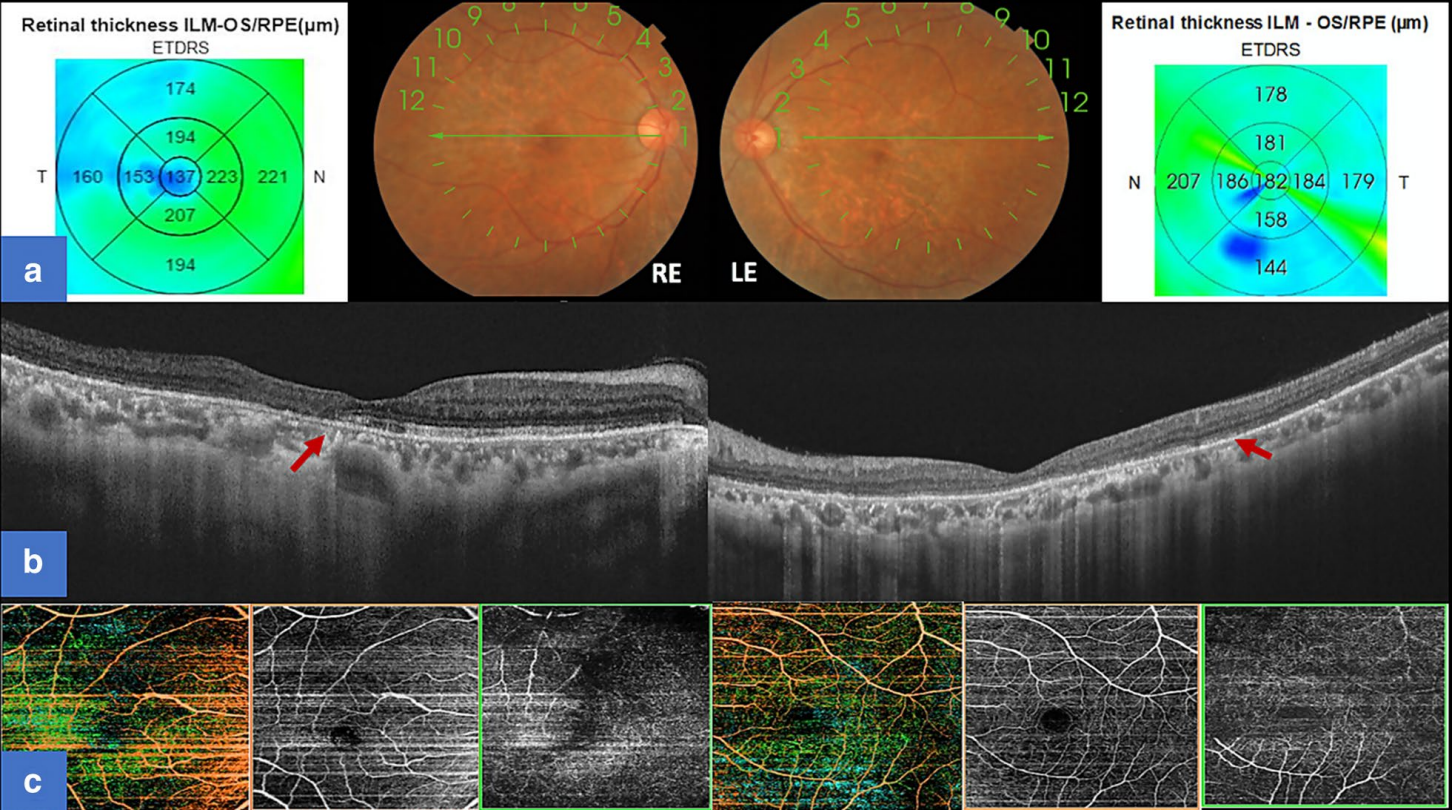
Optical Coherence Tomography-Angiography (OCT-A) of both eyes. **a** Panoramic color picture of the posterior pole, and the thickness macular map of right and left eye respectively. **b** B-Scan on the foveal line that shows a disruption of the external layers and photoreceptors (The *red arrow* shows the site of the disruption) of right and left eye respectively. **c** OCT-A that shows a composite angiography (*color image*), angiography of the superficial (*orange border*) and deep layer (*green border*) of the right and left eye. Shows a normal vascular pattern in the choriocapillaries, superficial and deeper vascular plexus

**Fig. 4:**
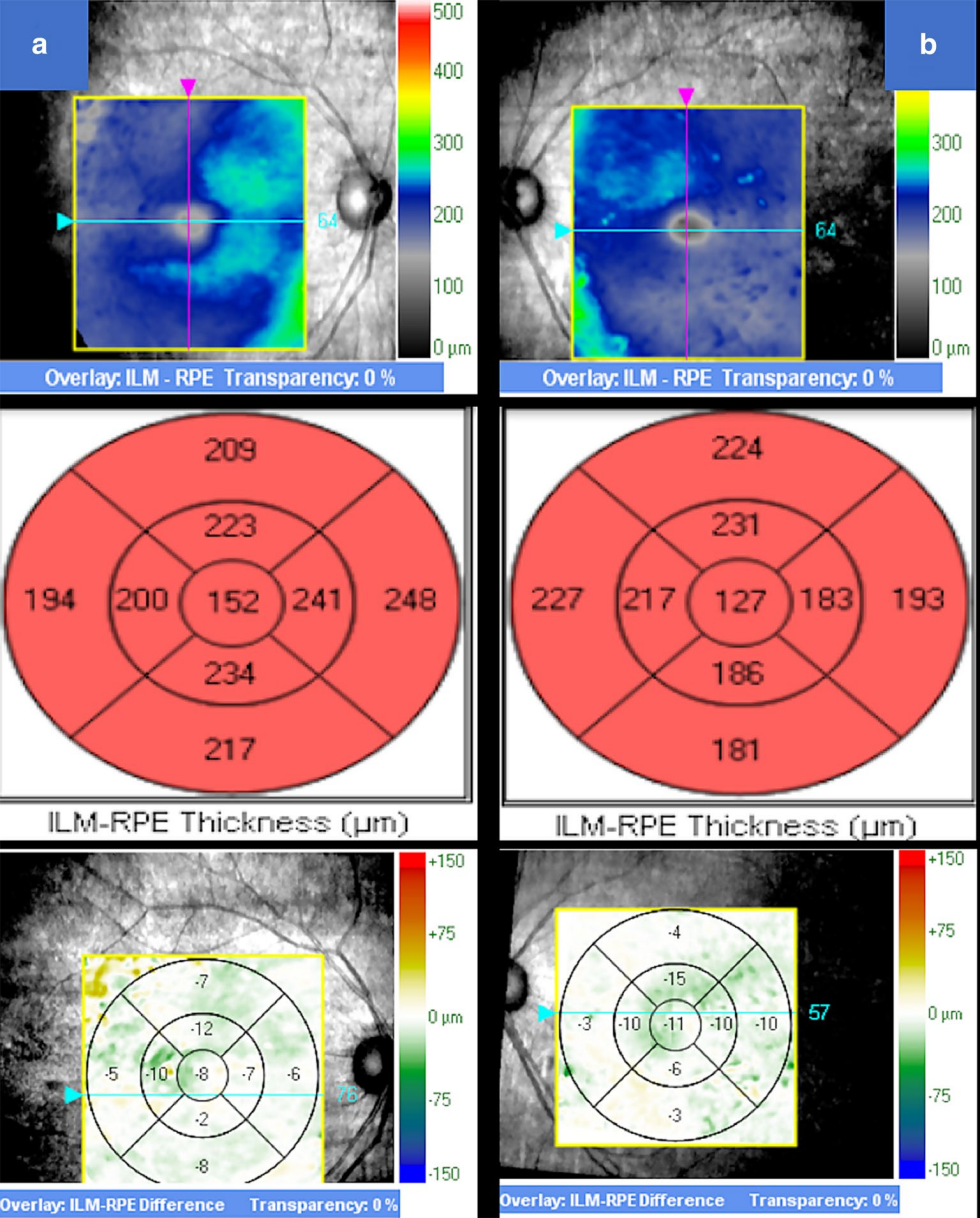
The HD SD OCT at 2 years’ follow-up revealed a mild decrease of the thickness at macular level in both eyes. **a** Right eye, **b** left eye

**Fig. 5:**
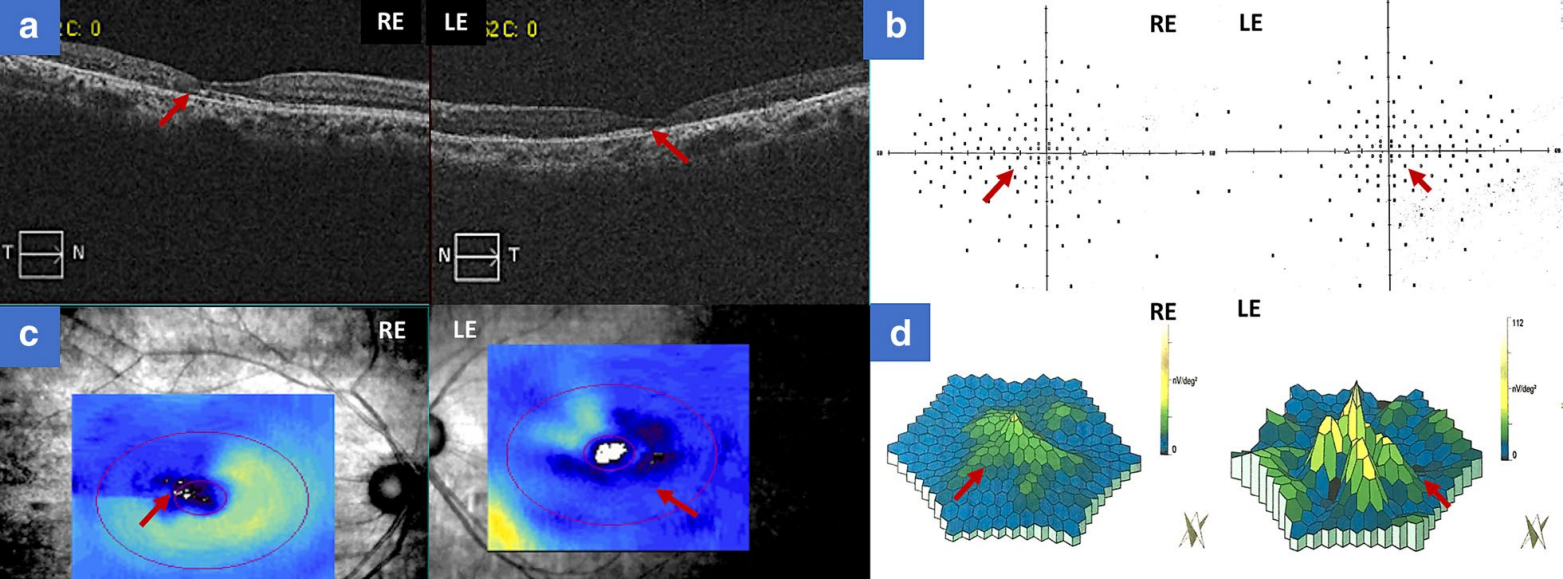
Anatomo Physiological correlation between OCT Cirrus 4000 scan, Neurological Visual Field and Multifocal Electroretinogram. **a** B-Scan of OCT Cirrus 4000 on foveal line of the right and left eye respectively (*Red arrow* shows the disruption of the photoreceptors). **b** Neurological visual field of both eyes (*Red arrow* shows the limit of the peripheral scotoma and mild macular respect). **c** Ganglion Cell Map of OCT Cirrus 4000 that shows a diffuse compromise of the ganglion cell with greater affection on the left eye. **d** Multifocal electroretinogram that shows a diffuse compromise with a mild macular respect in both eyes. (*Red arrow* shows the limit of the functional and dysfunctional retina at macular level)

**Fig. 6:**
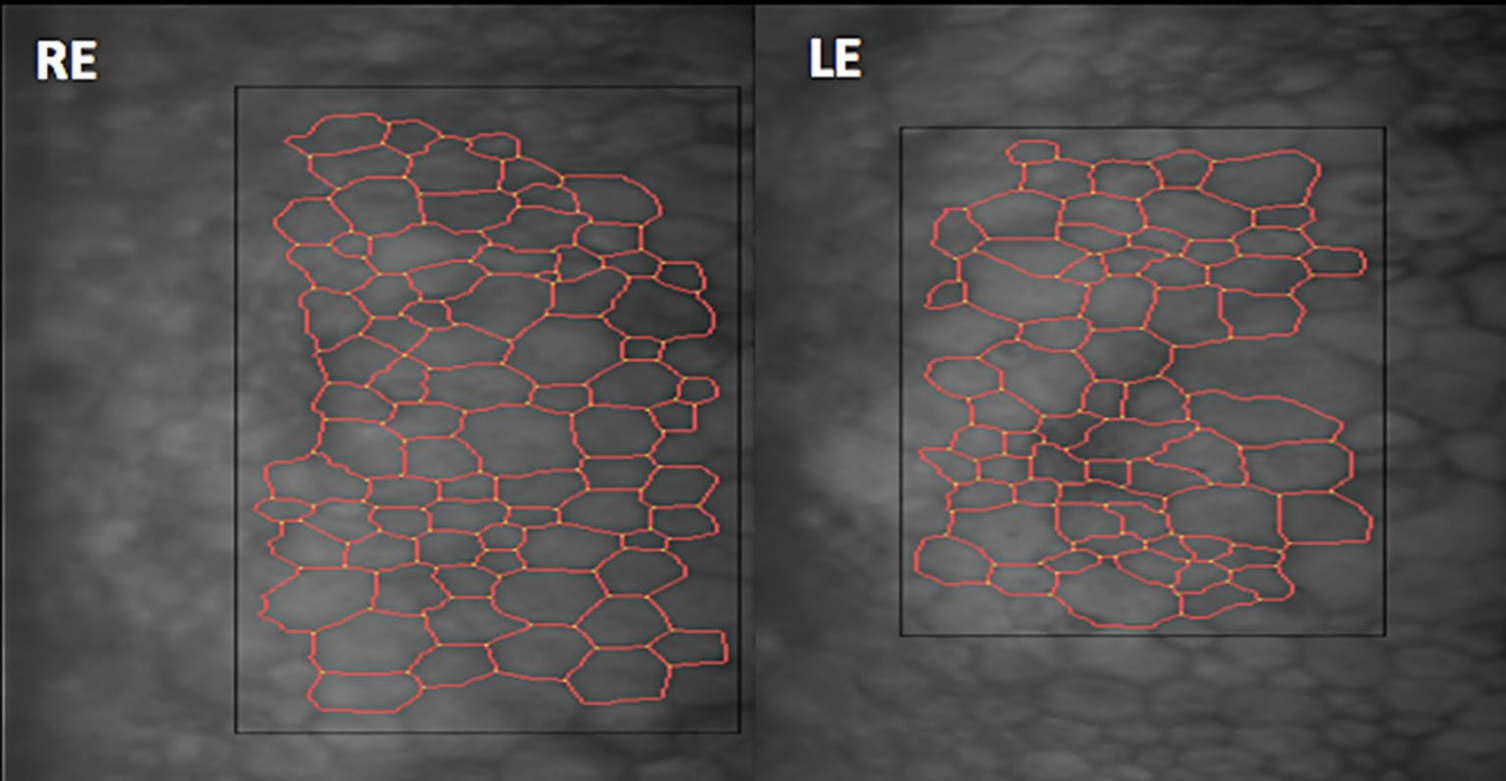
Specular microscopy shows the endothelial density with polymegathism and pleomorphism in both eyes

**Fig. 7:**
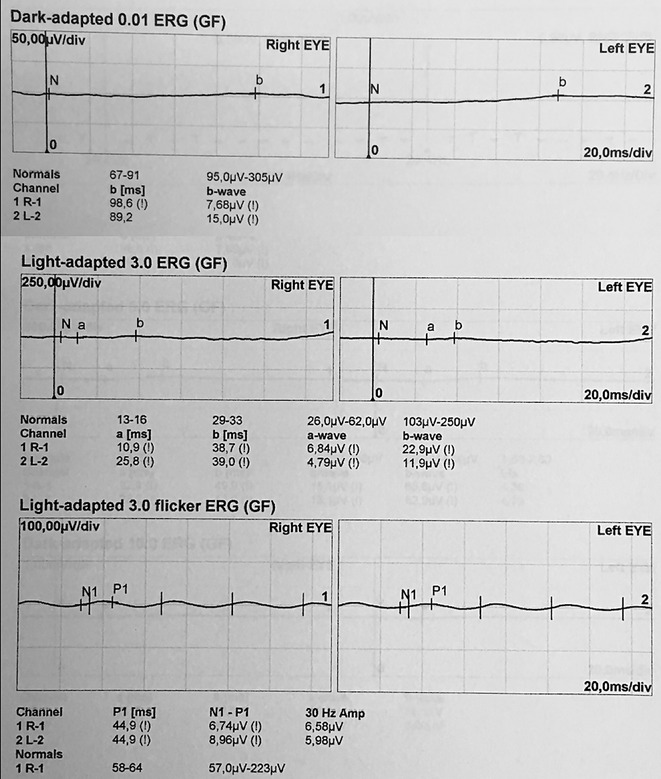
Full field electroretinogram that evidenced a compromise of the rod and cones with a subnormal level in the scotopic response with a borderline photopic response

A 55-year-old female with a history of controlled arterial hypertension, presented to the Retina and Neuro Ophthalmology service at FOSCAL (Bucaramanga, Colombia), reporting a history of 13 years of progressive decrease of visual acuity OU, bilateral ptosis and ophthalmoplegia. She had no remarkable ocular or systemic past history. Best corrected visual acuity (BCVA) in both eyes (OU) was 20/50. Asymmetric bilateral ptosis was found with generalized limitation of extraocular movements in OU with an exotropia in primary position of the gaze (Fig. 1), The cornea showed some none confluent guttae in OU and mild cataract both eyes. Fundus exam showed a normal optic nerve with atrophic and RPE changes with a salt and pepper appearance (Fig. 2). Fluorescein angiography (FA) showed areas of hyperfluorescence and hypofluorescence in a mottled pattern. The HD SD OCT (Carl Zeiss Cirrus 4000 HD OCT) revealed areas of outer retinal layer atrophy in OU, predominantly temporal to the fovea, with a disruption of the ellipsoid zone in mottled pattern in OD, severe diffuse asymmetric macular thinning and areas of RPE hyperplasia without changes in choroid. OCT-A (DRI OCT-1 Triton Topcon Japan) showed a normal vascular pattern in the choriocapillaries, superficial and deeper vascular plexus (Fig. 3). At 2 years’ follow-up with HD SD OCT (Carl Zeiss Cirrus 4000 HD OCT) showed a mild decrease of the macular thickness (Fig. 4) and a decrease of the ganglion cell OU (Fig. 5a–c). Specular microscopy showed a decrease of endothelial cell density (OD: 1526 cel/mm^2^ and OS: 1779 cel/mm^2^) with mild polymegathism and pleomorphism (Fig. 6). The computerized visual field of OU showed a diffuse depression of retinal sensitivity in OU with peripheral predominance (Fig. 5b). The electrophysiological studies evidenced a compromise of the rod and cones with a subnormal level in the scotopic response with a borderline photopic response in the full field electroretinogram (ERG) (Fig. 7) and multifocal ERG corroborated with the computerized visual field (Fig. 5d). Cardiological assessment showed electrocardiographic abnormalities compatible with left branch block of the bundle of Hiss (LBBB) and also signs of right ventricular hypertrophy. The serum lactate level was 4.6 mg/dL (normal values: 0.75–3.1). Myasthenia gravis and dysthyroid ophthalmopathy were discarded. The muscular biopsy showed polygonal fibers with peripheral placed nuclei, “Ragged Red fibers” were observed on Gomori Trichrome staining. Genetic consultation was done; no genetic testing was performed. With all these features, the clinical and histopathologic diagnosis of KSS was made.

## Discussion

KSS is a neuromuscular disorder caused by mitochondrial dysfunction, secondary to deletions of mtDNA [2, 3, 6, 7] ranging from 1000 to 10,000 DNA nucleotides. The most common deletion is 4.9 kb. A smaller proportion of cases are due to mtDNA duplications that can be transmitted through maternal inheritance [6]; in which impaired mitochondria are abnormally large and abundant in tissues of high metabolic consumption like skeletal muscles, myocardium, central nervous system and the RPE [4, 7]. These mitochondria abnormalities have been verified by Gomori trichrome stain and observed as “Ragged Red” fibers in pathological examination [7, 8]. It is for this reason (an alteration in mitochondria) that tissues with a high metabolic demand for energy and oxygen, especially at the ocular level, are frequently affected, such as corneal endothelium, extraocular muscles, pigment epithelium, among others organs; explaining the high range of systemic manifestations such as CPEO, palpebral ptosis, pigmentary retinopathy, reduced corneal endothelium count [9, 10].

The correlation between ophthalmoplegia and retinal pigmentary changes were described by Barnard and Scholtz in 1944 [11], later in 1946 Sandifer, document the association between ophthalmoplegia and cardiomyopathy suggesting the importance of the ocular muscle biopsy for the diagnostic [12], until in 1958 Kearn and Sayre made the description of the syndrome [1, 13]. The decrease of the visual acuity depends on the degree of retinal deterioration. The histopathological examination indicate that affectation predominates in the periphery of the retina rather than the posterior pole. In order of affection, RPE is initially affected, followed by changes in photoreceptors and choriocapillaris [4, 7, 8, 11–13]. The alterations of RPE and photoreceptors are generally atypical and diffuse, described as “Salt and Pepper” appearance in the most of the cases, but this changes can be highly variable [14, 15]. Among the different presentations described: areas of hyperpigmentation and hypertrophy of the RPE, chorioretinal degeneration and some fluffy white patchy lesions, even some authors like Ascaso et al. [15], reported a macular lesion resembling adult-onset vitelliform macular dystrophy and in other cases it could present with subretinal fluid [16]. All of these RPE changes are evidenced in histologic studies [4, 7, 8, 11–13], and these histopathological features are consistent with the findings in our patient evidenced in the HD SD OCT, so it is possible that with new OCT technology anatomical changes of outer retina layers in KSS can be seen consistently, specially with the use of enhanced depth imaging (EDI OCT) and OCT-A. Our patient doesn’t have a retinal histopathological study, but tomographical findings correlate with expected histopathological changes [7], where we see areas of RPE atrophy and hypertrophy, with some mottled areas of disrupted photoreceptors.

## Conclusion

We report a patient with unusual late onset of the KSS, accompanied by some alterations of the RPE and photoreceptors observed in the external HD SD OCT and OCT-A. These findings have a significant correlation with histopathological studies performed previously in other cases. In the best of our knowledge, this is the first report in the literature of HS SD OCT and OCT-A findings in a patient with KSS.

## Abbreviations

KSS: Kearns–Sayre syndrome
OCT: Optical Coherence Tomography
OCT-A: Optical Coherence Tomography-Angiography
HD SD OCT: High Definition Spectral Domain Optical Coherence Tomography
mtDNA: mitochondrial deoxyribonucleic acid
CPEO: chronic progressive external ophthalmoplegia
PCR: polymerase chain reaction
BCVA: best corrected visual acuity
OD: right eye
OS: left eye
OU: both eyes
RPE: retinal pigmentary epithelium
FA: fluorescein angiography
NFL: nerve fiber layer
ERG: electrorretinogram
LBBB: left branch block of the bundle of hiss
EDI OCT: enhanced depth imaging Optical Coherence Tomography
